# Patient-Reported Outcomes of Depression and Fibromyalgia Symptoms Do Not Predict Non-Inflammatory versus Inflammatory Diagnoses at Initial Rheumatology Consultation

**DOI:** 10.3390/healthcare12191948

**Published:** 2024-09-29

**Authors:** Arne Schäfer, Magdolna Szilvia Kovacs, Axel Nigg, Martin Feuchtenberger

**Affiliations:** 1 Medizinische Klinik und Poliklinik II, University Hospital Würzburg, 97080 Würzburg, Germany; schaefer@diabetes-zentrum.de; 2 Diabetes Zentrum Mergentheim, 97980 Bad Mergentheim, Germany; 3 Rheumatologie, MVZ MED BAYERN OST, 84489 Burghausen, Germany; magdolna.kovacs@med-bayern-ost.de (M.S.K.); axel.nigg@med-bayern-ost.de (A.N.)

**Keywords:** rheumatology, rheumatoid arthritis, fibromyalgia, patient-reported outcome measures, electronic health records, diagnosis, depression, pain

## Abstract

Objective: The objective of this study was to assess the potential value of patient-reported outcomes (PROs) of depression, fibromyalgia symptoms, and pain in predicting non-inflammatory vs. inflammatory diagnoses in rheumatology patients. Methods: This retrospective, single-center study evaluated electronic health record (EHR) data from adults who were seen for their first rheumatology consultation and subsequently received a diagnosis of an inflammatory (e.g., rheumatoid arthritis or spondyloarthritis) or non-inflammatory (e.g., osteoarthritis or fibromyalgia) condition. The PROs evaluated included depressive symptoms (Patient Health Questionnaire-2 [PHQ-2]), fibromyalgia symptom severity (FM SS), and pain. Results: A total of 3669 patients were evaluated, including patients with (n = 984; 26.82%) and without (n = 2685; 73.18%) inflammatory rheumatologic disease, of whom 141 (3.8%) had fibromyalgia. The non-inflammatory subgroup reported higher FM SS scores, and the inflammatory subgroup had higher pain and inflammatory markers. Bivariate models based on PHQ-2 and FM SS had a very low specificity (0.3%) for predicting non-inflammatory conditions, resulting in the misclassification of >99% of inflammatory cases. Adding pain, inflammatory markers, and other relevant EHR variables increased specificity but still resulted in a high level of misclassification. Conclusions: The PROs evaluated in this study are not suitable for predicting non-inflammatory vs. inflammatory rheumatologic disease, even when combined with other EHR variables.

## 1. Introduction

One of the most common reasons for patients to see a rheumatologist is musculoskeletal pain [1]. In Germany [2,3], as in many other countries [4,5], the demand for rheumatology care exceeds the available capacity. One of the main reasons for the long waiting times for initial rheumatology consultations is the high proportion of patients with non-inflammatory conditions who present for initial consultations; approximately three-quarters of patients referred to rheumatologists are diagnosed with a non-inflammatory condition [1]. Multiple studies have shown that the early diagnosis and treatment of inflammatory diseases, such as rheumatoid arthritis (RA) and spondyloarthritis, is of critical prognostic importance: it can prevent irreversible structural damage to joints and/or organs and preserve long-term function [6,7,8,9]. Although non-inflammatory disorders such as osteoarthritis (OA) and fibromyalgia (FM) are also treated by rheumatologists [10], in the setting of limited resources, a delay in the management of these conditions may be an acceptable trade-off in order to allow for the prompt treatment of inflammatory conditions.

Against this background, early arthritis consultations, patient questionnaires, and models for triage or prioritization prior to rheumatological investigations have been established in recent years [11,12,13,14,15,16,17]. The common goal is to give patients with immunological inflammation priority access to rheumatology care, thus keeping the diagnostic and therapeutic “window of opportunity” open. These approaches provide some assistance in identifying patients with inflammatory versus non-inflammatory disorders but generally lack the specificity required for a clinically useful screening tool or require advanced testing or imaging that may not be uniformly performed [15].

Although musculoskeletal symptoms are clearly a key element in diagnosing non-inflammatory versus inflammatory rheumatologic disorders, these are not always easy for patients and general physicians to identify [18,19]. In addition to musculoskeletal issues, patients often report other symptoms such as headaches, fatigue, reduced motivation, depressed mood, and pain [20,21]. Such symptoms could conceivably be related to a wide range of conditions, including primary pain disorder, secondary pain generalization in primary degenerative conditions, somatoform disorders, and inflammatory diseases [22,23,24].

Of the many potential diagnoses for patients with musculoskeletal symptoms, one of the most difficult to evaluate is FM as it shares symptoms with inflammatory conditions and occurs concomitantly with inflammatory arthritis in a substantial proportion of patients, including approximately 20% of patients with RA [25,26]. Characteristic FM symptoms include chronic widespread musculoskeletal pain often accompanied by depression and anxiety [26]. According to the American College of Rheumatology’s diagnostic criteria, the most important diagnostic variables for FM include widespread pain and the severity of somatic symptoms, including fatigue, waking unrefreshed, and cognitive symptoms [27]. Because these symptoms are also common in other patients, including those with inflammatory arthritis, FM is sometimes considered a “diagnosis of exclusion” [25].

This study examines the utility of electronic health record (EHR) data likely to be collected by general practices prior to rheumatology referral in predicting a non-inflammatory vs. inflammatory diagnosis in patients with musculoskeletal symptoms. Our primary focus is patient-reported outcomes (PROs) such as depressive mood, FM symptoms, and pain burden, which can be easily and systematically collected prior to referral, but we also examine other variables likely to be recorded in the EHRs, such as inflammatory markers. For the purposes of this study, inflammatory conditions include diagnoses such as RA, spondyloarthritis, vasculitis, and connective tissue disease, while the group of non-inflammatory patients is defined by the exclusion of such diagnoses and included FM.

Other studies have suggested that patient-reported outcomes (PROs) may be useful in triage approaches for rheumatology referrals [15], but to date, there is minimal information on differences in PROs between non-inflammatory and inflammatory conditions, and the available literature is primarily confined to evaluations of subsets of patients, such as patients with FM and RA, within these broader categories. Available data suggest that patients with FM have higher mean levels of FM symptoms, pain, and depression than patients with RA, but there is wide variability among patients, and the standard deviations for these two groups overlap [28,29]. The goal of this study is therefore to achieve a better understanding of whether PROs may be useful in predicting a subsequent non-inflammatory vs. inflammatory diagnosis in patients with musculoskeletal symptoms.

## 2. Materials and Methods

### 2.1. Study Design and Objectives

This retrospective cohort study aimed to investigate the differences in PRO symptomatology—specifically depression/mood alterations, fibromyalgia symptoms, and pain burden—between patients with non-inflammatory versus inflammatory underlying conditions, as diagnosed by a rheumatologist, and the ability of these outcomes to predict a non-inflammatory or inflammatory diagnosis. Data for this study were retrospectively collected from EHRs of patients 18 years of age or older who presented in person to a single, large secondary care center specializing in rheumatology and related disorders between 1 January 2020 and 31 December 2023 and received a documented primary diagnosis of an inflammatory condition (e.g., RA or spondyloarthritis) or exclusion of an inflammatory diagnosis due to a non-inflammatory condition (e.g., OA or FM) as defined by International Classification of Diseases (ICD)-10 codes. There were no additional inclusion or exclusion criteria. Following a rheumatologic assessment, patients for whom inflammatory rheumatic diseases were ruled out were referred to specialists in other disciplines for further evaluation.

### 2.2. Ethical Approval

This study was approved by the Institutional Review Board of Würzburg University with a waiver for individual patient consent given the retrospective design and use of de-identified patient data (#207/21-me). All research activities were conducted in accordance with the ethical principles outlined in the Declaration of Helsinki.

### 2.3. Measurement of Primary and Secondary Variables

The primary variables compared between subgroups in this study were PROs captured through questionnaires and tools. Depressive symptoms were evaluated using the 2-item Patient Health Questionnaire (PHQ-2), an established instrument that quantitatively assesses the extent of depressed mood over the past 2 weeks on a scale ranging from 0 (no depression) to 6 (severe depression) [30]. The FM symptom severity (SS) questionnaire was used to evaluate symptoms associated with FM, including waking unrefreshed, cognition, and fatigue, on a scale ranging from 0 (no symptoms) to 12 (severe symptoms) [27]. Pain burden was measured via the visual analog scale (VAS) for pain (pain [VAS]), which allows patients to express the level of pain intensity on a continuum ranging from 0 (no pain) to 100 (worst possible pain). Similarly, the VAS for the impact of disease on global activity as assessed by the patient (PtGA [VAS]) and physician (PhGA [VAS]) was utilized to assess patient and physician perceptions of the overall impact of disease.

Several secondary variables were also examined to provide a more in-depth overview of each patient’s health status, including the inflammatory markers C-reactive protein (CRP) and erythrocyte sedimentation rate (ESR). Vitamin D and thyroid-stimulating hormone (TSH) levels were measured through blood tests, offering insights into metabolic and endocrine functions that could influence symptoms. Additionally, body mass index (BMI) and sociodemographic variables, including age and gender, were collected to analyze their potential association with the primary outcomes and to ensure a comprehensive understanding of the study population.

### 2.4. Statistical Analysis

Sample size calculations were performed based on an anticipated small effect size of 0.2 (Cohen’s d) for the difference in FM SS values between patients with non-inflammatory vs. inflammatory diagnoses, an alpha level of 0.05, and a statistical power of 0.8. Our calculations indicated that a total sample of at least 788 participants (394 per subgroup assuming equal distribution) was required to detect significant differences between groups. These considerations regarding the optimal sample size provide an ample margin for asymmetric sample distributions, as subsequently observed in our data, and for the application of multivariate inferential statistical methods.

Initial exploratory data analysis involved summary statistics to describe the study population and the prevalence of reported symptoms. Chi-square tests and independent *t*-tests were applied to compare categorical and continuous variables, respectively, across the inflammatory and non-inflammatory groups. To further investigate the association between the type of underlying condition and symptom severity, binary logistic regression analyses were conducted. We chose ten predictors based on theoretical considerations and their clinical potential for association with a final non-inflammatory vs. inflammatory diagnosis, specifically morning stiffness, pain (VAS), PtGA (VAS), PHQ-2, FM SS, body mass index, ESR, CRP, gender, and age. The “ENTER” option in SPSS was used to avoid excluding potential predictors solely on statistical grounds. Receiver operating characteristic (ROC) curves and area under the curve (AUC) calculations were used to assess the diagnostic predictive ability of identified variables. Power analysis was performed a priori using G*Power software Release 3.1.9.6 to determine the optimal sample size. Data were analyzed using SPSS Statistics version 26 (IBM Corp., Armonk, NY, USA) and R software version 4.0 (R Foundation for Statistical Computing, Vienna, Austria).

## 3. Results

**Figure 1:**
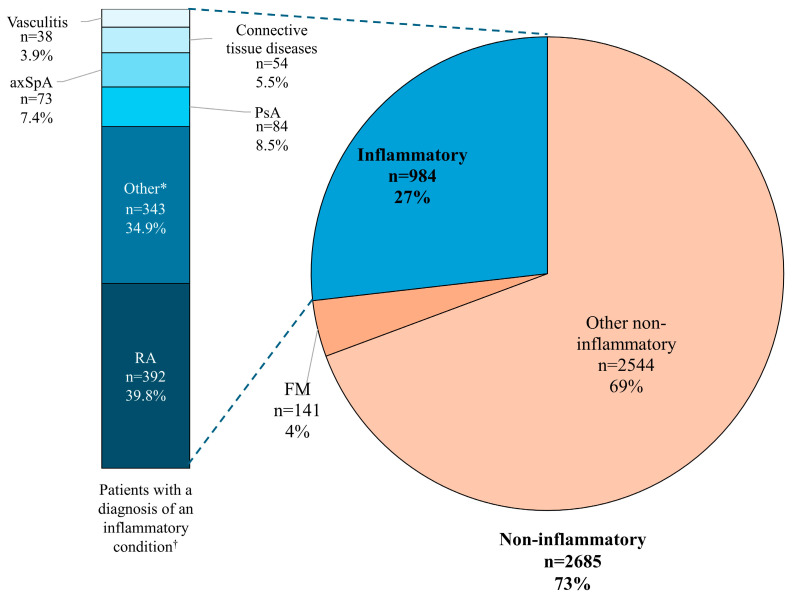
A diagnostic overview of the patient cohort. * Including diagnoses classified as other inflammatory arthritis conditions and other inflammatory conditions (e.g., polymyalgia rheumatica). ^†^ Ten patients in the inflammatory group had a diagnosis of FM (RA = 3; axSpA = 3; PsA = 2; other = 2). axSpA, axial spondyloarthritis; FM, fibromyalgia; PsA, psoriatic arthritis; RA, rheumatoid arthritis.

This retrospective analysis included a total of 3669 patients seen for a first consultation by a rheumatologist. Females accounted for 64.19% of the patient cohort (n = 2355), and the mean (standard deviation) age was 54.6 (15.3) years (range, 18.6 to 93.8 years). Of these patients, 2685 (73.18%) received a primary diagnosis of a non-inflammatory condition, and 984 (26.82%) received a primary diagnosis of an inflammatory condition as defined by ICD-10 codes (Figure 1). Because categorization was based on primary diagnoses, none of the patients were considered to have both a non-inflammatory and inflammatory condition for the purpose of these analyses. The most common inflammatory diagnosis was RA (n = 392; 10.68% of the total patients and 39.83% of patients with a diagnosis of an inflammatory condition) (Figure 1). FM was diagnosed in 151 patients, including 141 patients diagnosed with a non-inflammatory condition (5.25% of the non-inflammatory subgroup) and 10 patients diagnosed with an inflammatory condition (1.02% of the inflammatory subgroup). Additional information on specific non-inflammatory diagnoses was not available in our dataset as these patients were referred to other specialists for assessment.

### 3.1. Patient Characteristics According to Non-Inflammatory/Inflammatory Diagnosis

**Figure 2:**
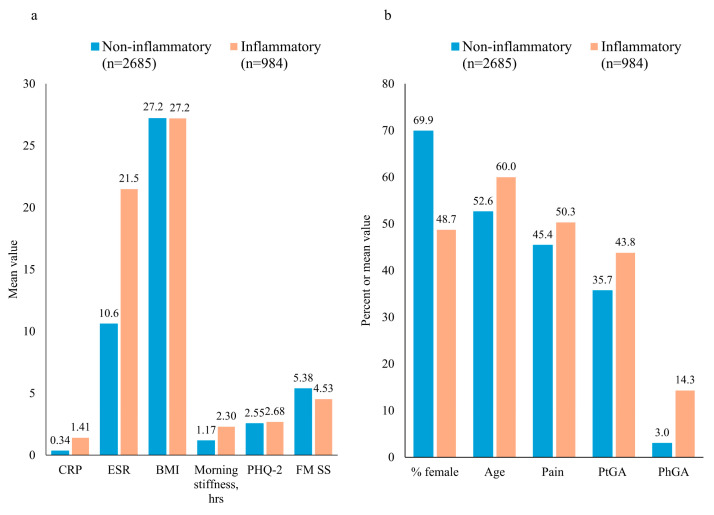
Differences between patients with non-inflammatory and inflammatory diagnoses in (**a**) mean CRP, ESR, BMI, morning stiffness, PHQ-2, and FM SS and (**b**) proportion of female patients according to mean age, pain (VAS), PtGA (VAS), and PhGA (VAS). Standard deviations are presented in Table 1. BMI, body mass index in kg/m^2^; CRP, C-reactive protein in mg/dL; ESR, erythrocyte sedimentation rate in mm/h; FM SS, fibromyalgia symptom severity; PHQ, Patient Health Questionnaire; PhGA, physician assessment of global disease activity; PtGA, patient assessment of global disease activity; VAS, visual analog scale.

The characteristics of the patients in the non-inflammatory and inflammatory subgroups as derived from EHRs are shown in Table 1 and Figure 2. As expected, the inflammatory subgroup had higher levels of inflammatory markers (CRP and ESR) as well as higher levels of morning stiffness, PhGA (VAS), PtGA (VAS), and pain (VAS). Moreover, patients in the inflammatory subgroup were older compared with those in the non-inflammatory subgroup (mean of 60.0 vs. 52.6 years). The non-inflammatory disease subgroup had a much higher proportion of females compared with the inflammatory subgroup (69.87% vs. 48.68%) and had higher mean FM SS scores (5.38 vs. 4.54) (Table 1).

For both the non-inflammatory and inflammatory subgroups, the PtGA (VAS) scores were markedly higher than the PhGA (VAS) scores, indicating that the patients considered their global disease activity to be greater than the physician’s assessment (Table 1 and Figure 2).

### 3.2. Prediction of Non-Inflammatory vs. Inflammatory Disease

**Figure 3:**
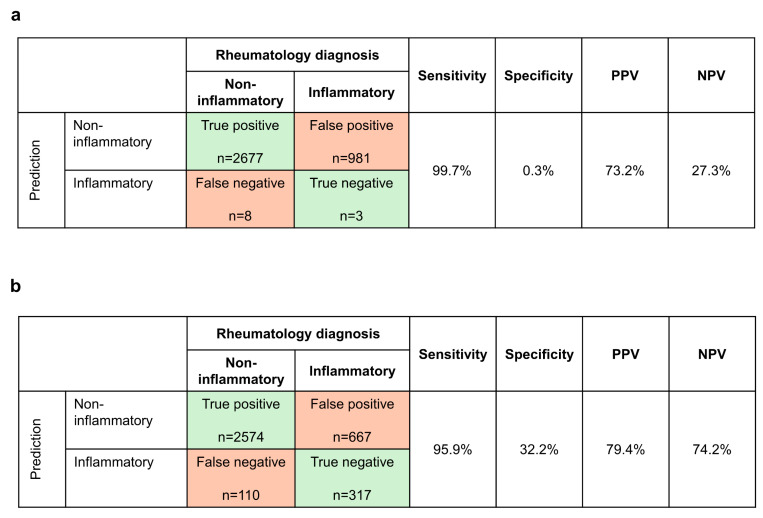
Predictive* ability for non-inflammatory conditions of (**a**) combination of PHQ-2 and FM SS and (**b**) 10-variable model including PHQ-2, FM SS, morning stiffness, pain (VAS), PtGA (VAS), BMI, CRP, ESR, female gender, and age. * Sensitivity = [True positives/(True positives + False negatives)] × 100; specificity = [True negatives/(True negatives + False positives)] × 100; PPV = [True positives/(True positives + False positives)] × 100; NPV = [True negatives/(True negatives + False negatives)] × 100. BMI, body mass index; CRP, C-reactive protein; ESR, erythrocyte sedimentation rate; FM SS, fibromyalgia symptom severity; NPV, negative predictive value; PHQ, Patient Health Questionnaire; PPV, positive predictive value; PtGA, patient global assessment of disease activity; VAS, visual analog scale.

We used a binary logistic regression analysis to evaluate whether non-inflammatory conditions in patients with musculoskeletal complaints could be predicted based solely on a combination of values from two PROs that were more likely to have higher values in non-inflammatory cases, the PHQ-2 and FM SS (Figure 3a). We found that the combination of PHQ-2 and FM SS had a high sensitivity (99.7%) for non-inflammatory conditions but a very low specificity (0.3%). In other words, it correctly identified 2677/2685 (99.7%) cases related to non-inflammatory conditions but incorrectly identified 981/984 (99.7%) cases of rheumatologist-diagnosed inflammatory disease as non-inflammatory conditions. The positive predictive value (PPV) was 73.2%, and the negative predictive value (NPV) was 27.3%. These numbers are reversed if the data are evaluated from the perspective of the correct identification of an inflammatory condition (PPV of 27.3% and NPV of 73.2%). Adding age and gender to the model resulted in minimal improvements in the classification results.

**Figure 4:**
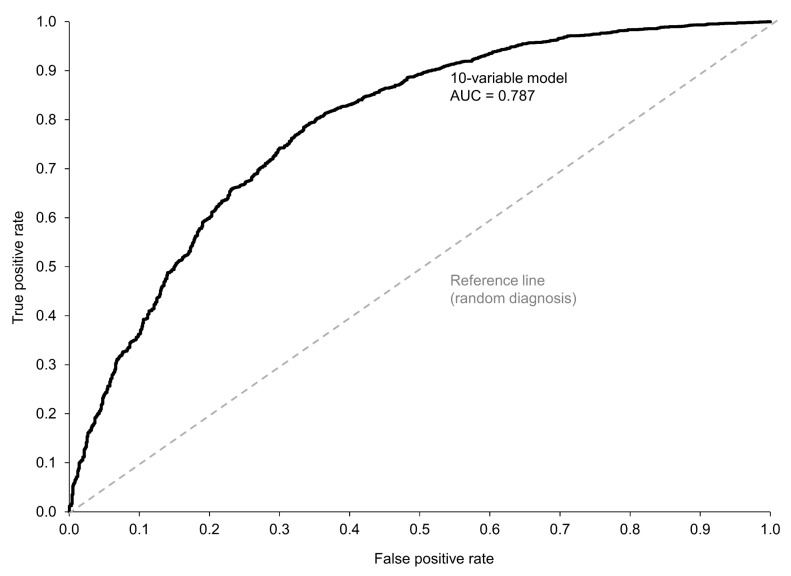
The receiver operating characteristic curve evaluating the diagnostic performance of the 10-variable model for a non-inflammatory condition as diagnosed by a rheumatologist. AUC, area under the curve.

We then expanded the model to encompass additional EHR data that could be relevant from a clinical perspective, including inflammatory markers and gender. The final prediction model included 10 variables: PHQ-2, FM SS, morning stiffness, pain (VAS), PtGA (VAS), BMI, CRP, ESR, female gender, and age (see Supplementary Table S1 for model parameters). This group of variables correctly identified 2574/2684 cases (95.9% sensitivity) with non-inflammatory disease and 317/984 cases (32.2% specificity) with inflammatory disease (one patient with a non-inflammatory diagnosis did not have ESR data and therefore was not included in this model) (Figure 3b). The addition of the EHR variables increased the NPV from 27.3% in the model with only PHQ-2 and FM SS to 74.2% in the 10-variable model and also resulted in a slight increase in the PPV (from 73.2% to 79.4%). The ROC curve for this model indicates that the diagnostic performance of the 10-variable model for a non-inflammatory condition is moderate (AUC = 0.787) (Figure 4).

## 4. Discussion

This study leveraged medical records from a total of 3669 patients with musculoskeletal symptoms to identify patterns that distinguished cases with non-inflammatory conditions from those with inflammatory disorders. In particular, our goal was to evaluate whether PROs might be useful in predicting a non-inflammatory vs. inflammatory diagnosis in patients presenting for an initial rheumatology consultation. We found that the combination of values for PHQ-2, a screening tool for depression, and FM SS, an assessment of FM symptoms, had a very low specificity (0.3%) for predicting underlying non-inflammatory conditions. Adding pain, inflammatory markers (CRP and ESR), the female gender, and other potentially relevant EHR variables substantially improved the model, but the low specificity of the resulting model prevents it from being clinically useful.

Ideally, routine tests or questionnaire data derived from EHRs would provide a framework for patients at an elevated risk for inflammatory disease to be automatically flagged and “fast-tracked” to undergo a rheumatology evaluation. Other studies have found that PROs can be highly useful in identifying inflammatory conditions [15], so we focused our attention on the PROs that might distinguish between non-inflammatory and inflammatory conditions. Unfortunately, the combination of assessments of depression and FM symptoms used in this study was not adequate for predicting non-inflammatory vs. inflammatory disease. The PPV of 73.2% was identical to the proportion of patients with rheumatologist-confirmed non-inflammatory disorders in this cohort, indicating that this combination of PROs does not provide a useful addition in predicting the ultimate diagnosis.

Expanding the model to include other key clinical variables improved its performance. The ROC AUC of 0.787 indicates that this expanded model had moderate diagnostic performance; typically, values of ≥0.7 are considered acceptable for diagnostic tests [31]. However, the specificity of the 10-variable model was only 32.1%, which is too low to be clinically useful and would leave the majority of patients with an inflammatory disorder misclassified as having a non-inflammatory condition, potentially further contributing to delayed treatment.

Our study is one of a number of attempts to identify clinical or PRO variables that could be clinically useful in differentiating non-inflammatory and inflammatory patient populations. As an example, the criteria developed by the European Alliance of Associations for Rheumatology (EULAR) to identify patients with clinically suspect arthralgia who were likely to develop RA had a PPV of 30% among patients who had been identified with arthralgia by a rheumatologist, but this value dropped to 3% in patients who had not had a rheumatology evaluation [32]. These criteria required a rheumatology assessment, which negates their usefulness as a diagnostic screening tool. Inflammatory markers, particularly CRP, have not been found to be useful predictors of RA, in part due to a lack of specificity [33,34]. Morning stiffness is also considered an inflammatory marker and is associated with early and subclinical RA [35,36]. Prolonged morning stiffness is likely to indicate an inflammatory condition, but it can also be a symptom of FM [37] and OA [38], which impairs its ability to accurately distinguish between inflammatory and non-inflammatory conditions. Finally, auto-antibody lab tests and imaging have shown utility in differentiating between inflammatory and non-inflammatory disorders [39,40,41,42,43], but a substantial proportion of patients with these features do not develop inflammatory arthritis, and the widespread use of these diagnostic tools can increase the burden on healthcare systems.

It is likely that the strong overlap between symptoms and characteristics in non-inflammatory and inflammatory conditions contributed to the suboptimal performance of these models. Depression is extremely common in FM, affecting up to 63% of patients at some point during their lives [44], but it is also common in patients with inflammatory arthritis [45] and is therefore not suitable for distinguishing between non-inflammatory and inflammatory subgroups. Similarly, pain and symptoms of FM covered by the FM SS questionnaire, such as fatigue, are characteristics of FM [27,46], but they are also shared by patients with inflammatory conditions [47,48]; in fact, the pain values were higher in patients with an inflammatory diagnosis versus those with a non-inflammatory diagnosis in our study.

Based on the reported PRO values, patients in both the non-inflammatory and inflammatory subgroups were experiencing a substantial amount of pain and disease activity impairment at their initial rheumatology consultation. The patient assessments of disease activity (PtGA [VAS]) were markedly higher than the physician assessments (PhGA [VAS]), indicating that the patients perceived their conditions to be more distressing than the physicians. This discordance between the PtGA and PhGA assessments has been previously reported for established RA and other types of inflammatory arthritis [49,50] and appears to be driven by several factors, most notably pain and fatigue [51,52,53]. Our data demonstrate that the discordance between PtGA and PhGA also extends to patients with early RA as well as those with other musculoskeletal complaints, including non-inflammatory rheumatology conditions.

The limitations of this study include the retrospective, single-center design. This study was designed to evaluate specific PRO variables rather than the full range of clinical and PRO characteristics. This approach allowed us to focus on data easily obtained from EHRs during routine clinical care rather than primarily confined to specialist medical care. Future studies should consider the inclusion of additional assessments of pain, such as the widespread pain index or the McGill Pain Questionnaire, as well as additional laboratory results, such as auto-antibodies, as predictive variables. The proportion of patients with FM was fairly small in this study (n = 151 [4.12%]), and 10 of the FM patients were classified as inflammatory cases. It is possible that co-morbid non-inflammatory and inflammatory conditions may have complicated the analyses of the predictive variables. In particular, FM occurs in approximately 13% to 21% of patients with inflammatory arthritis [25]. However, this is also a challenge faced in rheumatology practice, so it provides an accurate reflection of predictive ability during clinical care. We do not have additional information on non-inflammatory diagnoses, as once a rheumatic inflammatory disorder was excluded, these patients were referred to other specialists for additional evaluations. One study of patients with non-inflammatory vs. inflammatory conditions found that the most common non-inflammatory diagnosis was osteoarthritis (40.7% of patients), followed by “other miscellaneous diagnoses” (38.1%) [12]. We suspect that our non-inflammatory patient population would have similar results. An important strength of our study is that the large number of included patients far exceeded the required sample size of 788, thereby providing a robust level of confidence in our results.

## 5. Conclusions

We found that the combination of depressive symptoms, as measured by the PHQ-2, and FM symptom severity, as measured by FM SS, was not useful in predicting rheumatologist-diagnosed non-inflammatory and inflammatory conditions in patients with musculoskeletal symptoms who had not yet been seen by a rheumatologist, even when combined with other EHR data. Accordingly, although the PROs of depression and FM symptoms are important for an overall assessment of patient health, clinicians should not rely on these to predict inflammatory or non-inflammatory diagnoses. Additional variables, such as the duration from symptom onset to appointment requests [54], or new approaches, such as the application of artificial intelligence [55], may be required to accurately distinguish between non-inflammatory and inflammatory conditions prior to an in-person assessment by a rheumatologist.

## Figures and Tables

**Table 1:** Clinical characteristics and PRO values based on inflammatory/non-inflammatory diagnosis. Data are reported as mean (standard deviation) unless otherwise specified.

Characteristic	Rheumatology Diagnosis	
	Non-Inflammatory (n = 2685)	Inflammatory (n = 984)
Female, n (%)	1876 (69.87)	479 (48.69)
Age, years	52.6 (14.9)	60.0 (15.3)
Body mass index, kg/m^2^	27.2 (5.8)	27.2 (5.2)
Morning stiffness, h	1.17 (3.5)	2.30 (5.3)
CRP, mg/dL	0.34 (0.67)	1.41 (2.32)
ESR, mm/h	10.6 (10.0) ^a^	21.5 (19.2)
Vitamin D, ng/mL	26.9 (14.0)	25.5 (12.7)
TSH, μU/mL	1.62 (1.10)	1.68 (1.20)
PhGA (VAS)	3.0 (7.1) ^b^	14.3 (16.9)
PtGA (VAS)	35.7 (28.4)	43.8 (30.9)
Pain (VAS)	45.4 (26.7)	50.3 (29.2)
PHQ-2	2.55 (1.84)	2.68 (1.88)
FM SS	5.38 (3.12)	4.53 (2.95)

^a^ n = 2684; ^b^ n = 2679. CRP, C-reactive protein; ESR, erythrocyte sedimentation rate; FM SS, fibromyalgia symptom severity; PHQ, Patient Health Questionnaire; PhGA, physician assessment of global disease activity; PtGA, patient assessment of global disease activity; TSH, thyroid-stimulating hormone; VAS, visual analog scale (0 to 100).

## Data Availability

The dataset is available upon request from the authors.

## Supplementary Materials

The following supporting information can be downloaded at https://www.mdpi.com/article/10.3390/healthcare12191948/s1, Table S1: Model parameters for the 10-variable model for prediction of a non-inflammatory versus inflammatory diagnosis.
