# Supplementary material for: Patient-Reported Outcomes of Depression and Fibromyalgia Symptoms Do Not Predict Non-Inflammatory versus Inflammatory Diagnoses at Initial Rheumatology Consultation
Source: Healthcare (Basel). 2024 Sep 29;12(19):1948. doi: 10.3390/healthcare12191948 (PMC11475572; doi:10.3390/healthcare12191948)
Supplement: Supplementary file 1 [file healthcare-12-01948-s001.zip › healthcare-3194249-supplementary.pdf]

# Supplemental Materials

## Patient-reported Outcomes of Depression and Fibromyalgia Symptoms Do Not Predict a Non-inflammatory versus Inflammatory Diagnosis at Initial Rheumatology Consultation

**Table S1**. Model parameters for the 10-variable model for prediction of a non-inflammatory versus inflammatory diagnosis.

| Model entry | Regression coefficient (beta) | Standard error | Wald test | Significance | Odds ratio[a] (95% CI) |
|---|---|---|---|---|---|
| **Variable** | | | | | |
| Morning stiffness | -0.031 | 0.10 | 10.491 | 0.001 | 0.969 (0.951, 0.988) |
| Pain (VAS) | 0.003 | 0.002 | 1.320 | 0.251 | 1.003 (0.998, 1.007) |
| PtGA (VAS) | -0.008 | 0.002 | 15.453 | <0.001 | 0.992 (0.988, 0.996) |
| PHQ-2 | -0.029 | 0.030 | 0.960 | 0.327 | 0.971 (0.917, 1.029) |
| FM SS | 0.089 | 0.017 | 26.275 | <0.001 | 1.093 (1.057, 1.131) |
| BMI | 0.027 | 0.008 | 11.408 | 0.001 | 1.027 (1.011, 1.043) |
| ESR | -0.033 | 0.004 | 55.639 | <0.001 | 0.968 (0.960, 0.976) |
| CRP | -0.479 | 0.064 | 56.484 | <0.001 | 0.619 (0.547, 0.702) |
| Female | 0.849 | 0.090 | 88.629 | <0.001 | 2.337 (1.958, 2.789) |
| Age | -0.018 | 0.003 | 35.526 | <0.001 | 0.983 (0.977, 0.988) |
| **Constant** | 1.378 | 0.274 | 25.212 | <0.001 | 0.252 |

All variables had one degree of freedom.

[a]Odds ratio is equivalent to exponent B and describes the predicted change in odds for a one unit change in the predictor.

BMI, body mass index; CI, confidence interval; CRP, C-reactive protein; ESR, erythrocyte sedimentation rate; FM SS, fibromyalgia symptom severity; PHQ, Patient Health Questionnaire; PhGA, physician assessment of global disease activity; PtGA, patient assessment of global disease activity; VAS, visual analog scale (0 to 100)
